# The Mechanisms for the Association of Cancer and Esophageal Dysmotility Disorders

**DOI:** 10.3390/medsci9020032

**Published:** 2021-05-21

**Authors:** Francisco Tustumi, Jorge Henrique Bento de Sousa, Nicolas Medeiros Dornelas, Guilherme Maganha Rosa, Milton Steinman, Edno Tales Bianchi

**Affiliations:** 1 Hospital Israelita Albert Einstein, Sao Paulo 05652-900, Brazil; jorgehbs@hotmail.com (J.H.B.d.S.); nmdornelas@hotmail.com (N.M.D.); guilhermemaganha@gmail.com (G.M.R.); milton.steinman@einstein.br (M.S.); ednotales@gmail.com (E.T.B.); 2 Department of Gastroenterology, Faculdade de Medicina, Universidade de Sao Paulo, Sao Paulo 05403-000, Brazil

**Keywords:** achalasia, esophagus, squamous cell carcinoma, carcinoma, neoplasm

## Abstract

Background: Achalasia and other esophageal dysmotility disorders mimicking achalasia can be associated with cancer. This study aimed to review the main mechanisms for which cancer may develop in esophageal dysmotility disorder patients. Methods: A narrative review was performed. Results: The mechanism for developing squamous cell carcinoma and adenocarcinoma are discussed. Besides, achalasia-like syndromes related to familial KIT-gene mutation and pseudoachalasia are discussed. Conclusions: Knowing the main mechanism for which achalasia can be related to cancer is essential for clinicians to conduct the proper investigation, surveillance, and treatment.

## 1. Introduction

Achalasia is an esophageal motor disease characterized by the lack of relaxation of the lower esophageal sphincter (LES) and aperistalsis [1]. The first time this condition was described was in 1672 by Sir Thomas Willis, but the “achalasia” term was only created in 1929 by Hurst and Rake, meaning “no relaxation” [2]. The main mechanism for which achalasia appears is due to neuronal degeneration of the myenteric plexus, although the reason for it to happen is still uncertain [2]. Reduced density of Cajal cells in the gastroesophageal junction, the gastrointestinal pacemakers, is another mechanism that may be related in some patients [3].

Achalasia is more often seen in South American countries due to its association with Chagas disease [4]. Patients with achalasia can initially show dysphagia, recurrent chest pain, regurgitation, and weight loss [5]. Usually, symptoms are nonspecific, and there is a significant delay in diagnosis, so the majority of the patients begin their treatment in advanced stages of the disease, already presenting malnutrition, underweight, and recurrent pneumonia [5]. Manometry is the gold-standard diagnostic test for achalasia. The typical findings are incomplete relaxation of LES and uncoordinated esophageal body contractions. Frequently, LES is hypertensive [6]. Barium swallow tests reveal a dilated esophagus, the reason for which achalasia is often called megaesophagus. Besides, barium swallow tests may reveal uncoordinated esophageal contractions and a narrowing at the gastroesophageal junction [7]. Patients with achalasia have an increased risk for developing esophageal cancer [8,9].

Fagge was probably the first author to point out a possible association between achalasia and cancer in 1872 [10]. He described a case of a benign esophageal condition associated with a tumor. Sato et al. [11], analyzing a sample size of 2714 achalasia patients, evidenced that the risk of developing cancer was 16.7 times higher for men with achalasia and 8.8 times higher for women with achalasia.

When patients start to have symptoms of cancer, the tumor is usually already advanced. The symptoms of achalasia and esophagus cancer are similar, and the patient may undervalue the symptoms. Additionally, esophageal-related symptoms depend on the esophagus distension and longitudinal contraction of esophageal smooth muscle, and in achalasia, the esophagus is naturally enlarged [12]. In this way, achalasia patients may present only mild esophageal symptoms, even with a severe condition within it [13]. For the carcinoma to overlap the symptoms of achalasia, it must be large to obstruct a dilated esophagus [14]. Due to the delay in the differential diagnosis, many patients end up having a late cancer diagnosis, and the locally advanced disease leads to poor prognosis of these patients, often making the curative treatment unfeasible [9].

Despite many studies establishing an association between achalasia and cancer, achalasia’s actual cancer genesis mechanisms are poorly debated. This study aims to review the literature on the pathophysiology of cancer development in achalasia and other esophageal dysmotility disorders mimicking achalasia.

## 2. Materials and Methods

A narrative review was performed, gathering the medical evidence regarding the mechanisms for which achalasia and other esophageal dysmotility disorders that mimic achalasia may be related to cancer development. We provided an overview of the available research evidence for clinicians to know the different neoplasm types that may be associated with esophageal dysmotility disorders. The following search terms were used: “achalasia”, “megaesophagus”, “esophageal dysmotility”, “esophageal motility disorders”, “dysphagia”, “esophageal cancer”, “neoplasms”, “pseudoachalasia”. The main databases searched were PubMed, Embase, Lilacs/BVS, Cochrane Central, and Google Scholar. The study design included any observational or experimental human study and animal models.

## 3. Results

The present review searched for the main evidence of the mechanisms involving esophageal dysmotility and cancer. They include epithelial neoplasms, such as squamous cell carcinoma and adenocarcinoma, and also mesenchymal cancers. Both esophageal and extra-esophageal cancer were discussed.

### 3.1. Esophageal Squamous Cell Carcinoma

The incidence of squamous cell carcinoma in achalasia is 312/100,000 patient-years at risk [9]. Although there is no controlled study comparing different geographic regions, South American cohorts report a higher incidence of cancer per patients with achalasia than in achalasia cohorts in the rest of the world [9]. This possible geographic variability in the incidence of esophageal squamous cell carcinoma may correlate to diet aspects. In fact, in non-achalasia patients, squamous cell carcinoma is related to nutritional and dietary habits, including vitamins, minerals, meats, salted foods, mycotoxins, and even hot beverages [15].

Beyond the diet, a large number of other factors may contribute to cancer. Continuous saliva stasis and food decomposition in the esophagus may induce chronic hyperplastic esophagitis [16]. Inflammation may contribute to cancer development through several different ways, including angiogenesis, DNA damage, promotion of cellular growth and multiplication, and dysregulation of programmed cell death [17]. The esophageal stasis also predisposes to recurrent lesions in the esophagus due to the increased time of exposure of the esophageal mucosa to substances such as alcohol, tobacco, and nitrosamines, leading to chronic inflammation. Additionally, the origin of the achalasia is immune-mediated, and organ neurons and ganglions are affected by cytotoxic T-cells, mast cells, eosinophils, and antibodies, contributing to a chronic inflammatory process [18].

Overgrowth of nitrate-reducing bacteria due to esophageal food stasis leads to increased volatile N-nitrosamines concentration in the esophageal lumen [19]. N-nitrosamines originated with nitric oxide reactions [20]. Overproduction of reactive nitrogen species (RNS) leads to nitrosative stress [20]. In this circumstance, there will be damage to the lipids, proteins, and nucleic acids [20], and consequently, lead to genotoxic and carcinogenic effects, as demonstrated in animal models [21]. RNS concentration can be increased by the excess of food intake or by endogenous production in the food stasis [21]. Esophageal achalasia has specific microbiota, which may vary according to the grade of dilatation [22]. Among the microorganisms found in the achalasia esophagus, *Staphylococcus* sp., *Corynebacterium* sp., *Peptostreptococcus* sp., and *Veillonella* sp. can produce N-nitrosamines [22]. N-nitrosamines under certain pH conditions decompose into alkyldiazhydroxide species, and the alkylation of the DNA produces modified purine and pyrimidine bases [23].

Achalasia predisposes to psychiatric conditions, probably due to its influence on patient well-being [24,25]. Psychiatric syndromes can predispose these patients to alcoholism [11,26], a known risk factor for esophageal squamous cell carcinoma [11]. Alcohol in stasis may lead to much more intense esophagitis, including acute necrotizing esophagitis [27].

Some studies investigated the genetic pathways in the development of cancer in achalasia. Munari et al. [28] reported an association between Chagasic achalasia and *PIK3CA* mutations. Patients with these mutations presented a significantly lower survival rate. *PIK3CA* encodes phosphoinositide 3-kinase (PI3K), an upstream kinase that regulates cell proliferation, apoptosis, and growth [29]. However, there is no certainty if these mutations are caused by the *Trypanosoma* infection or by chronic esophageal dilatation.

In another study investigating genetic changes in esophageal cancer related to Chagasic achalasia, Lacerda et al. [30] showed a high rate of *TP53* mutations in these cancers, but there was no difference comparing with non-achalasia squamous cell carcinoma. Safatale-Ribeiro et al. [31] showed that *TP53* overexpression and mutational changes, including exons 5, 6, and 7, are more often seen in patients with high-grade inflammation achalasia of the esophagus. In addition to the *TP53* gene mutation, *p21, p16*, and epidermal growth factor receptor may also play a role in the carcinogenesis in achalasia [16].

Patients with Chagasic achalasia have also a higher prevalence of chromosome aneusomies, including chromosomes 7, 11, and 17 [32]. These cytogenetic alterations are known to predispose patients to squamous cell carcinoma [33].

Finally, Chagasic achalasia squamous cell carcinoma patients also present a higher rate of microsatellite instability than non-achalasia squamous cell carcinoma (21% vs. 11%) [34]. This finding may point to future immunotherapy options for these patients since microsatellite instability is a surrogate prognostic biomarker for immunotherapy in other cancer types [35].

### 3.2. Esophageal Adenocarcinoma

While the carcinogenesis of squamous cell carcinoma in achalasia has been well investigated in previous studies, so far, there are few studies evaluating achalasia and the development of esophageal adenocarcinoma. In a national Swedish inpatient data evaluation, both esophageal squamous cell carcinoma and adenocarcinoma were increased in achalasia patients [36]. The incidence rate of esophageal adenocarcinoma is 21/100,000 achalasia patient-years at risk [9]. However, the incidence probably varies across different worldwide geographic regions [11]. Nonetheless, the mechanisms for which adenocarcinoma develop in achalasia are poorly understood.

There is a possibility that the esophageal achalasia environment acts as a cancer risk for both types of esophageal carcinoma, and that for some reason, oncogenic events may end up in squamous cell carcinoma or adenocarcinoma. Both acid reflux clearance impairment and chronic inflammation due to food stasis within the esophagus may act as carcinogenesis mechanisms for squamous cell carcinoma and adenocarcinoma [37]. As well as for squamous cell carcinoma, the bacteria metabolites, such as N-nitrosocompounds, lactic acid, and heme, have an association with Barrett’s esophagus-adenocarcinoma sequence [38,39]. In addition to the nitrosative stress, the oxidative stress in chronic esophagitis induces replacement of the stratified squamous epithelium for a metaplastic columnar epithelium, leading to Barrett’s esophagus and, eventually, adenocarcinoma [40]. In fact, achalasia associated with Barrett’s esophagus has been previously reported [41].

Besides, achalasia’s main therapeutic approaches aim to perform LES disruptures, such as myotomy or endoscopic dilation. Those procedures can contribute to gastroesophageal reflux and induce metaplastic transformation of squamous mucosa to Barrett’s esophagus [36]. In this sense, caution is needed in patients submitted to peroral endoscopic myotomy (POEM) without anti-reflux procedures. Patients with achalasia have limited esophageal sensibility to esophagitis, and often POEM patients present high-grade esophagitis with only mild or no symptoms. [13,42].

The precise mechanism for which Barrett’s esophagus appears after chronic oxidative stress is not fully understood. Chronic esophagitis promotes increased metabolism of molecular oxygen, raising the intracellular concentration of reactive oxygen species (ROS). ROS can act at several steps in multistage carcinogenesis, participating in numerous signaling cascades, inducing both initiation and progression of cancer. Most of the mutations related to ROS involves base pair substitution, and all nucleobases can be affected, producing mispaired DNA sequences [43]. The 8-OH-deoxyguanosine (8-OHdG), a biomarker of oxidative stress, is known to be elevated in both Barrett’s esophagus and esophageal adenocarcinoma [44]. In addition, the superoxide dismutase and the glutathione redox system, which acts to mitigate the oxidative stress insult, are reduced in Barrett’s esophagus tissue. [45]. Long-standing elevated ROS activate transcription factors, such as activator protein-1 (AP-1) and nuclear factor-kappaB (NF-κB). NF-κB translocates into the nucleus, where it binds to promote a target oncogene. Alternatively, upstream kinases activated by both oxidative and pro-inflammatory stimuli activate AP-1 components, promoting transcriptional activation of oncogenes, leading to cell proliferation and growth [46]. Additionally, pro-inflammatory cytokines and microRNAs have a significant role in adenocarcinoma development. Barrett’s esophagus shows mutations even before the cancer development [47]. A cumulative number of mutations is the underlining mechanism for the sequence of Barrett’s esophagus to adenocarcinoma [37]. These genetic changes include *TP53* and *P16* expression, copy number alterations, and altered gene transcription patterns [37]. The overexpression of *TP53* and numeric abnormalities of chromosome 7 that are often seen in achalasia patients [32] are also known to be critical cytogenetic biomarkers for adenocarcinoma precursors [48].

Non-achalasia studies of adenocarcinomas without evidence of Barrett epithelium hypothesize that the neoplasm raised from heterotopic gastric mucosa or gastric mucosa extends into the esophagus or even esophageal glands. [49,50]. It is known from *Helicobacter pylori* and autoimmune gastritis studies that chronic inflammation increases the risk for developing gastric adenocarcinoma, and it could apply to the heterotopic gastric mucosa or esophageal glands [51]. Consequently, chronic esophagitis in achalasia is a presumable risk variable for malignization, even with no Barrett epithelium evidence. In addition, it is known that gastroesophageal reflux induces chronic inflammation and oxidative stress in the cardia and proximal gastric mucosa, and thus, procedures that disrupt the LES and favor the gastric reflux may contribute to proximal gastric cancer development [52].

### 3.3. Other Types of Cancer

In addition to the epithelial cancers, other achalasia-associated neoplasms have already been described, although rare.

Interstitial cells of Cajal (ICC) are present in the gastrointestinal wall and act as pacemakers, coordinating the gastrointestinal organs’ motility [53]. Malfunctioning of these cells may lead to an achalasia-like phenotype.

ICC expresses high levels of KIT (CD117) in their surface, also known as stem cell growth factor receptor [54]. KIT is a type III tyrosine kinase receptor and is expressed in several normal tissues, including melanocytes, mast cells, hematopoietic stem cells, germ cells, basal cells, and some cells in the nervous system. This receptor is encoded by the proto-oncogene *c-KIT*. Animal models have shown a link between *c-KIT* mutation and a low ICC density, leading to gastrointestinal dysmotility [55].

Besides, ICC are the progenitor cells of gastrointestinal stromal tumors (GISTs), and a gain-of-function mutation of the *c-KIT* may lead to these tumors [56]. The tyrosine kinase activity induces phosphatidylinositol 3-kinase and mitogen-activated protein kinase pathways [57]. There are several reports of familial achalasia-like and GISTs. Hirota et al. [58] reported familial gastrointestinal stromal tumors with achalasia-like symptoms and *c-KIT* mutation. These patients showed a single base pair mutation in the extracellular domain of stem cell gene mutation. Hoshino et al. [59] described two patients with familial achalasia, and one of them had gastric GIST.

Mast cells also highly express tyrosine kinase receptors on their surface, and thus, *KIT*-gene mutation may also induce mastocytosis [56]. Halpern et al. [60] investigated seven members of a family with achalasia-like symptoms. The patients had a unique activating mutation in exon 9 of *c-KIT*. All of the patients had mastocytosis, and three of them also developed GISTs. Marshall et al. [61] evaluated a family’s pedigree spanning four generations with achalasia-like symptoms and mastocytosis. Other findings in this family were diffuse esophageal leiomyomatosis, urticaria pigmentosa, neurofibroma, and mastocytosis.

The association of the KIT and the KIT ligand, also known as stem cell or mast cell growth factor, allows the signaling for the correct development, growth, migration, and differentiation of the melanocytes [62]. The association between pigment cell anomalies and *c-KIT* mutation is well established, including certain types of melanomas, such as acro lentiginous melanoma [63]. Neuhann et al. [64] reported a novel c-*KIT* mutation in family members presenting achalasia-like symptoms. The familial members had an autosomal dominant genetic disorder due to germline mutation in exon 11 of the *c-KIT*. All of them had multiple hyperpigmented skin macules, and some of the members also presented multiple GISTs. Ávila et al. [65] also reported a *c-KIT* mutation in exon 11 in an Argentinian family. Patients presented dysphagia associated with GISTs, diffuse melanosis, and lentiginosis. In a large kindred of 22 members with the *c-KIT* mutation, the index patient had esophageal thickening and was diagnosed with multiple smooth muscle tumors, dying within the 2 months follow-up. At the time, there was no genetic mutation suspected. The second family member called the medical staff’s attention for a possible inheritance disorder. This patient had multifocal low-grade leiomyosarcoma and hyperpigmentation of the perineum, hands, knees, and in the surrounding area of the mouth. The patient was submitted to a genomic study from leukocytes, confirming mutation in exon 11 of the *KIT-gene* [66] Other studies of patients with dysphagia and gastrointestinal stromal tumors or mast or melanocyte disorders linked with the *c-KIT* mutation have been reported [67,68,69,70]. In these cases, exons 8 [71], 9 [60], 11 [64,65,66,70], 13 [67], and 17 [58,69] mutations of the *c-KIT* were described.

*PDGFRA* is another type III tyrosine kinase receptor. The *PDGFRA* activates when binding to PDGFs and elicits the same pathways of the KIT signaling [72]. Consequently, mutations of PDGFRA hypothetically could lead to the same phenotype of the *c-KIT* mutation [72]. PDGFRA mutation syndromes account for a minority of the GISTs [72]. However, no *PDGFRA* mutations related to achalasia-like have been described yet.

Knowledge of the *c-KIT* mutation in these neoplasms opens a wide field to explore target therapy studies in the future. The use of tyrosine kinase inhibitors, such as Glivec (STI571; Imatinib. Novartis, Basel, Switzerland), was found to promote downstaging and metastasis control of GIST with *c-Kit* expression [73]. The low molecular weight tyrosine kinase inhibitor Imatinib blocks activation of KIT by binding the normal ATP binding site. [73]. A pooled analysis from ten European Organisation for Research and Treatment of Cancer (EORTC) databases [74] evaluated neoadjuvant Imatinib in advanced GISTs and showed excellent long-term survival outcomes and facilitation to resection. Most of the patients were preoperatively evaluated for *c-KIT* and *PDGFRA* mutations. In a recent randomized clinical trial [75], long-term use of Imatinib as adjuvant therapy for GIST improved the recurrence-free survival rates. In an open-label, non-controlled study [76], unresectable melanoma patients with a proven *c-KIT* mutation were evaluated for Imatinib response, demonstrating notable radiographic improvement. In a systemic mastocytosis study [77], Imatinib showed a 50% response, including 40% of sustained complete response. Ávila et al. [65] demonstrated an expressive response of the melanosis and gastrointestinal stromal tumors in the family members with achalasia-like symptoms. Moreover, tyrosine kinase inhibitors may change the Cajal cell’s pacemaker activity in the gastrointestinal system [78]. Halpern et al. [60] reported in their study that three of the patients were treated with tyrosine kinase inhibitors for GIST and mastocytosis. They noted robust response to the drug, with reduction of GIST size, reduction of bone marrow mast cells, and interestingly, patients revealed an expressive improvement in dysphagia.

Table 1 summarizes the main studies evaluating families’ pedigrees with germline *c-KIT* mutation, achalasia-like symptoms, and cancer.

### 3.4. Cancer Leading to Esophageal Dysmotility

Dysphagia mimicking achalasia may also be secondary to neoplasms instead of the other way around. Pseudoachalasia is a condition in which symptoms, manometric, endoscopic, and barium swallow test findings are pretty similar to achalasia. The first description of pseudoachalasia was in 1919 by Howarth [79]. He discussed a case report of esophageal dilatation with no anatomical stenosis as a manifestation of malignancy.

Pseudoachalasia is caused by malignancy in the majority of patients [80]. Esophageal, gastric, and esophagogastric junction neoplasms account for 46% of the causes of pseudoachalasia [80]. Following, lung (13%) and breast (6%) cancer are the most common causes of pseudoachalasia in non-gastrointestinal tumors [80]. Non-malignant causes are less frequent and include post laparoscopic gastric band, post vagotomy, descending aortic aneurysm, post sleeve gastrectomy, and stricture at the gastrojejunostomy [80].

The mechanism by which pseudoachalasia occurs can be due to direct tumor invasion and compression of the LES, or local nerves infiltration, leading to LES dysfunction. Moreover, the mass effect may block food transit and lead to progressive dilatation of the esophagus [81]. This is the main mechanism by which adenocarcinoma of the gastroesophageal junction leads to pseudoachalasia. Fabian et al. [82] reported pseudoachalasia as the first manifestation of a cardia adenocarcinoma in a patient that presented typical signs of achalasia at endoscopy, manometry, and the barium swallow test.

Besides, paraneoplastic pseudoachalasia may happen by the host’s immune response against tumor antigens, which leads to neuropathy [83]. It happens both by activation of immunocytes and by humoral response [84]. Most of the patients with neuronal paraneoplastic pseudoachalasia express serum autoantibodies, and the most common is the antineuronal nuclear antibody type-1 (ANNA-1; or Anti-Hu) [85]. Anti-acetylcholine and anti-neural calcium channel antibodies may be present in some patients as well [82,86]. Previous in vitro studies showed that these antibodies bind to neuronal antigens. The inflammatory/immune insult to the myenteric plexus could explain esophageal dysmotility [86].

A neuronal autoimmune disorder is more often associated with small-cell carcinoma, which in most cases arises in the lung [87]. Antigens from the small-cell carcinoma induce B-lymphocyte and helper T-lymphocyte responses. The first descriptions of neuronal autoimmune disorder in small-cell carcinoma were related to sensory neuronopathy and encephalomyeloradiculoneuropathy, but several other neurological conditions have already been reported, such as cerebellar ataxia, limbic encephalitis, myelopathy, Lambert–Eaton syndrome, and myopathy [87].

Immunohistochemical studies showed that ANNA-1 are RNA-binding proteins that act mainly in the nucleus of neurons in both the peripheral and central nervous systems. All myenteric ganglia may be affected, and ANNA-1 has been linked to other gastrointestinal tract dysmotility, such as gastroparesis, megacolon, and intestinal pseudo-obstruction [84]. Liu et al. [85] described a patient with severe dysphagia and a metastatic lung small cell carcinoma. Sera analysis showed circulating ANNA-1 IgG. Analysis of the specimens from myotomy showed fibrosis and perineural and intraneural lymphocytic infiltration, mainly CD8-positive T cells, and a paucity of myenteric ganglia. Interestingly, this finding resembles the degeneration of the myenteric ganglions in the primary achalasia.

For the correct differentiation of typical achalasia from pseudoachalasia, clinicians need to assess symptoms onset and manometric findings. Duration of symptoms is much shorter in pseudoachalasia than primary achalasia (median duration 13 vs. 36 months) [88]. Besides, achalasia patients are usually younger and have less severe weight loss than pseudoachalasia patients. In esophageal manometry, lack of intact peristalsis is less frequent in pseudoachalasia than in primary achalasia. Additionally, integrated relaxation pressure and esophagogastric junction contractile integral are significantly lower in pseudoachalasia than primary achalasia. [88]

The treatment of pseudoachalasia is focused on finding and treating the primary etiology. Endoscopic dilatation and botulinum toxin injection usually do not provide dysphagia relief [72]. Hirano et al. [89] reported two lung cancer patients with dysphagia. These two patients had advanced cancer and were treated with gastrostomy as palliative therapy.

Other studies also reported pseudoachalasia associated with cancer [90,91,92,93,94,95,96,97,98,99,100,101,102,103,104,105,106,107,108,109,110,111,112,113,114,115,116,117,118,119,120,121,122]. Table 2 summarizes the main case series and case reports of patients with pseudoachalasia caused by malignancy.

## 4. Discussion

**Figure 1:**
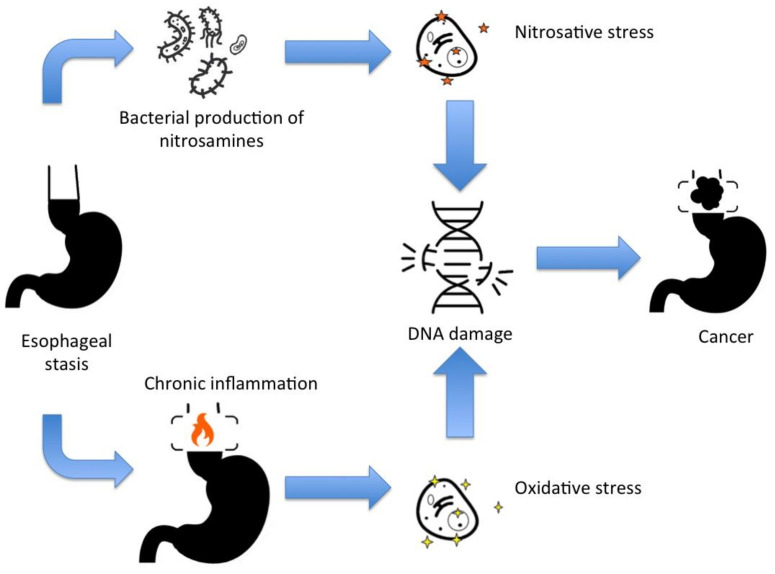
The main mechanisms for DNA damage and cancer development in achalasia.

Achalasia is a risk factor for developing cancer, including squamous cell carcinoma and adenocarcinoma. Figure 1 schemes the mechanism for DNA damage and mutations for cancer development in achalasia. Additionally, other esophageal dysmotility disorders mimicking achalasia symptoms may be associated with other malignant-related conditions, such as paraneoplastic manifestations and familial *KIT*-gene mutation.

Other review studies have also issued the cancer development in achalasia. A previous meta-analysis tried to investigate the incidence of cancer in achalasia [9,123]. However, due to the relatively low incidence of achalasia, the exact incidence of cancer in achalasia is still obscure once the risk for publication and selection biases are more common for small sample-sized studies. [124]. Torres-Aguilera et al. [125] pointed the main links between achalasia and cancer. The present review attempted to provide comprehensive rationality for clinicians to understand all the mechanisms for esophageal dysmotility disorders that are cancer-related. In fact, some patients will present with only dysphagia, and clinicians should be aware of the risk of associated neoplasms and should know the principal differential diagnosis, and how to investigate, follow, surveil, or treat.

In this sense, we can highlight some take-away lessons. Firstly, patients with idiopathic or Chagasic achalasia should be under surveillance for esophageal cancer since the risk for cancer progressively increases with the time for follow-up [9]. Surveillance should be continued even if patients are appropriately treated with myotomy or esophageal endoscopic dilation since there is no current evidence that these procedures reduce the risk for carcinogenesis [9]. Under a surveillance program, patients have a higher chance of early cancer diagnosis, which is amenable for endoscopic resection [11]. In achalasia patients, chromoendoscopy may help to upgrade the accuracy of endoscopic surveillance [126]. Additionally, dysphagia worsening in achalasia should always be actively investigated for cancer.

Esophagectomy would be the unique treatment that would null the risk for malignant transformation in achalasia patients. However, esophagectomy is related to a high risk for morbidity and mortality. Esophagectomy for cancer is associated with a risk of 36% of postoperative complications, and a 30-day postoperative mortality rate of 6.7% [127].

Another take-away lesson is that clinicians need to remember that more than one member of the same family with achalasia-like symptoms, mast cell or skin pigmentation disorders, or GISTs should be investigated for *c-KIT* mutation. As well, patients with acute onset achalasia-like symptoms with severe weight loss or dysphagia in the elderly should call attention for pseudoachalasia. Additionally, dysphagia in non-gastroesophageal cancer should always be investigated for a paraneoplastic neuronal disorder.

However, there are still more questions than answers in the field of cancer and esophageal dysmotility. Future studies are needed to fill the medical literacy gaps. Future studies should investigate the interventions by which clinicians could reduce the risk for squamous cell carcinoma or adenocarcinoma development in achalasia. There is no evidence that myotomy or endoscopic dilatation could reduce the mechanisms for achalasia malignization [7], and controlled studies are needed to investigate it. Additionally, there is a concern regarding the long-term results of POEM. Will long-term follow-up of POEM patients show a higher risk for progressing to Barrett-adenocarcinoma sequence?

Reactions involving both ROS and N-nitrosamines are potential targets for cancer chemoprevention in achalasia. Drugs that potentially mitigate the oxidative and nitrosative stress may help not only unveil future prophylaxis for achalasia-related cancer but also Barrett’s-related cancer. An animal model study evaluated the use of NG-nitro-L-arginine methyl ester (L-NAME), a NOS inhibitor, for cancer therapy. The study demonstrated that NOS inhibitors reduced the tumor growth rate [128]. Other preclinical studies using L-NAME or a similar arginine analog have shown similar results in the tumor growth rate [129,130].

Theoretically, dietary antioxidants could act to prevent oxidative stress insults in the esophageal food stasis. Curcumin, resveratrol, epigallocatechin gallate, caffeic acid phenethyl ester, isothiocyanates are antioxidative phytochemicals that could be evaluated for cancer prevention [46].

Additionally, antimicrobial therapy for changing the esophageal stasis microbiota theoretically could reduce the colonization of bacteria that can produce N-nitrosamines.

In addition, the studies of neoplasm biomarkers, such as microsatellite instability, may point to future new therapies, including immunotherapies, for patients with achalasia-related carcinoma. Additionally, tyrosine kinase inhibitor studies may show the benefit of treating certain types of esophageal dysmotility and *c-KIT* mutation syndromes.

## 5. Conclusions

Achalasia is associated with cancer. Knowing the main mechanisms for which achalasia can be related to cancer is essential for clinicians to conduct the proper investigation, surveillance, and treatment. Besides, it may help to enkindle future studies that may provide specific interventions for cancer prophylaxis and treatment in achalasia.

## Figures and Tables

**Table 1:** Germline c-KIT mutation associated with achalasia-like symptoms and neoplasms development. Main studies.

Author	Number Affected of Family Members	*c-KIT* Mutation	GIST	Mastocytosis	Other Neoplasm	Skin Hyperpigmentation
Ávila et al.	11	exon 11	4	0	0	8
Hartmann et al.	3	exon 8	2	3	0	0
Hirota et al.	6	exon 17	6	0	0	0
Hoshino et al.	2	not evaluated	1	0	0	0
Halpern et al.	7	exon 9	3	7	0	0
Neuhann et al.	3	exon 11	2	0	0	3
Marshall et al.	14	not evaluated	0	2	Leiomyomatosis (4); Neurofibroma (5)	14
Robson et al.	22	exon 11	14	0	Leiomyosarcoma (2)	12
Yamanoi et al.	1	exon 13	1	0	0	0
Vilain et al.	9	exon 13	4	0	0	3
O’Riain et al.	5	exon 17	5	1	Metastatic hepatic nodule (1)	1
Neves et al.	1	exon 11	1	0	0	0

GIST: Gastrointestinal stromal tumor.

**Table 2:** Pseudoachalasia secondary to malignancy. Main studies.

Author	N	Probable Mechanism	Neoplasm
Fabian et al.	1	Direct LES invasion	Adenocarcinoma of GEJ
Hirano et al.	2	Paraneoplastic	Lung cancer
Wenzl et al.	1	Direct LES invasion	Adenocarcinoma of GEJ
Eustace et al.	1	Extrinsic compression	Rhabdomyosarcoma
Nazareno et al.	1	Paraneoplastic	Breast cancer
Campo et al.	10	Direct LES invasion, extrinsic compression	Adenocarcinoma of GEJ, gastric adenocarcinoma, mediastinal tumor, pancreatic cancer, lung cancer
Saino et al.	1	Direct LES invasion	Pleural mesothelioma
Portale et al.	2	Direct LES invasion	Pancreatic cancer, breast cancer
Pastor et al.	1	Extrinsic compression	Lymphoma
Moorman et al.	1	Extrinsic compression	Lymphoma
Paulsen et al.	1	Direct LES invasion	Metastatic breast cancer
Lahbabi et al.	1	Direct LES invasion	Metastatic breast cancer
García-Alonso et al.	1	Direct LES invasion	Pancreatic cancer
Anaizi et al.	1	Direct LES invasion	Metastatic lung cancer
Bholat et al.	1	Direct LES invasion	Metastatic cervical cancer
Haberstroh et al.	1	Direct LES invasion	Bladder cancer
Branchi et al.	1	Direct LES invasion	Pleural mesothelioma
Campos et al.	2	Direct LES invasion	Gastric cancer, lung cancer
Rebollo et al.	1	Extrinsic compression	Lymphoma
Then et al.	1	Extrinsic compression	Lymphoma
Ulla et al.	3	Direct LES invasion, paraneoplastic	Prostate cancer, bladder, vocal cord cancer
Ter et al.	1	Direct LES invasion	Esophageal adenosquamous carcinoma
Agrusa et al.	1	Direct LES invasion	Adenocarcinoma of GEJ
Song et al.	3	Direct LES invasion	Adenocarcinoma of GEJ
Borst et al.	1	Direct LES invasion	Pancreatic cancer
el-Newihi et al.	1	Direct LES invasion	Gastric cancer
Stone et al.	1	Direct LES invasion	Esophageal adenocarcinoma
Moonka et al.	1	Direct LES invasion	Adenocarcinoma of GEJ
Liu et al.	13	Direct LES invasion, paraneoplastic	Adenocarcinoma of GEJ, esophageal adenocarcinoma, pleural mesothelioma, breast cancer, renal cell carcinoma, lung cancer
Choi et al.	1	Direct LES invasion	Metastatic liver cancer
Hejazi et al.	1	Paraneoplastic	Lung cancer
Kotoulas et al.	1	Direct LES invasion	Esophageal mesenchymal tumor
Iascone et al.	1	Extrinsic compression	Gastric cancer
Leung et al.	1	Paraneoplastic	Cholangiocarcinoma
Hsu et al.	1	Direct LES invasion	Gastric cancer
Lawal et al.	1	Direct LES invasion	Esophageal adenocarcinoma

## Data Availability

The data that support the findings of this study are available from the corresponding author, [F.T.], upon reasonable request.
